# Gray Matter Volume and Functional Connectivity in Hypochondriasis: A Magnetic Resonance Imaging and Support Vector Machine Analysis

**DOI:** 10.3389/fnhum.2020.596157

**Published:** 2020-12-02

**Authors:** Zhe Shen, Liang Yu, Zhiyong Zhao, Kangyu Jin, Fen Pan, Shaohua Hu, Shangda Li, Yi Xu, Dongrong Xu, Manli Huang

**Affiliations:** ^1^Department of Psychiatry, The First Affiliated Hospital, College of Medicine, Zhejiang University, Hangzhou, China; ^2^The Key Laboratory of Mental Disorder’s Management of Zhejiang Province, Hangzhou, China; ^3^Zhejiang Engineering Center for Mathematical Mental Health, Hangzhou, China; ^4^Department of Anesthesiology and Pain, The Affiliated Hangzhou First People’s Hospital, Zhejiang University School of Medicine, Hangzhou, China; ^5^Key Laboratory for Biomedical Engineering of Ministry of Education, Department of Biomedical Engineering, College of Biomedical Engineering and Instrument Science, Zhejiang University, Hangzhou, China; ^6^Columbia University and New York State Psychiatric Institute, Riverside Drive, New York, NY, United States

**Keywords:** hypochondriasis, gray matter volume, functional connectivity, support vector machine, default mode network

## Abstract

**Objective**: Patients with hypochondriasis hold unexplainable beliefs and a fear of having a lethal disease, with poor compliances and treatment response to psychotropic drugs. Although several studies have demonstrated that patients with hypochondriasis demonstrate abnormalities in brain structure and function, gray matter volume (GMV) and functional connectivity (FC) in hypochondriasis still remain unclear.

**Methods**: The present study collected T1-weighted and resting-state functional magnetic resonance images from 21 hypochondriasis patients and 22 well-matched healthy controls (HCs). We first analyzed the difference in the GMV between the two groups. We then used the regions showing a difference in GMV between two groups as seeds to perform functional connectivity (FC) analysis. Finally, a support vector machine (SVM) was applied to the imaging data to distinguish hypochondriasis patients from HCs.

**Results**: Compared with the HCs, the hypochondriasis group showed decreased GMV in the left precuneus, and increased GMV in the left medial frontal gyrus. FC analyses revealed decreased FC between the left medial frontal gyrus and cuneus, and between the left precuneus and cuneus. A combination of both GMV and FC in the left precuneus, medial frontal gyrus, and cuneus was able to discriminate the hypochondriasis patients from HCs with a sensitivity of 0.98, specificity of 0.93, and accuracy of 0.95.

**Conclusion**: Our study suggests that smaller left precuneus volumes and decreased FC between the left precuneus and cuneus seem to play an important role of hypochondriasis. Future studies are needed to confirm whether this finding is generalizable to patients with hypochondriasis.

## Introduction

Hypochondriasis is a disabling and exhausting psychiatric disorder, which can be characterized by unexplainable beliefs and a fear of having a lethal disease. Individuals with hypochondriasis typically would check for health-related information online frequently, which is regarded as the major cause leading to the occurrence and development of hypochondriasis (Starcevic and Berle, 2013). Recent surveys revealed a growing proportion of individuals affected by hypochondriasis ranging from 3.4% in the Australian population (Sunderland et al., 2013) to a surprising 20% of outpatients in medical clinics in London (Tyrer et al., 2011). These patients may spend excessive time and money seeking repeated medical examinations and diagnosis from medical specialists. Meanwhile, a large proportion of patients experience comorbid psychiatric distress, with anxious, depressive, and somatoform symptoms. Nevertheless, investigators still debate the neurological status and conceptualization of hypochondriasis. The diagnosis of hypochondriasis was recently updated by two new concepts, somatic symptom disorder (SSD) and illness anxiety disorder (IAD), in the Diagnostic and Statistical Manual of Mental Disorders, 5th edition (DSM-5; Wise and Birket-Smith, 2002; Olatunji et al., 2009). In contrast, hypochondriasis was moved from the category of somatoform disorders in the International Classification of Disease (ICD)-10 to those of Obsessive–Compulsive and Related Disorders in ICD-11 (Stein et al., 2016; van den Heuvel et al., 2014). Hence, exploring hypochondriasis is of great importance in understanding its biological mechanism. In this article, we report our study based on a group of patients with hypochondriasis recruited locally, which hopefully may provide novel perspectives for the neuroimaging findings related to pathogenic mechanisms of hypochondriasis.

Gray matter (GM) abnormalities have been associated with the clinical behavior of psychiatric disorders, including decreased GM volume (GMV) in the left middle frontal gyrus (MFG), which is likely due to the depressive mood in post-stroke depression (Hong et al., 2020), and reduced GMV in obsessive–compulsive disorder (Valente et al., 2005). Some structural magnetic resonance imaging (sMRI) studies have demonstrated a distinct dysfunction in the GM of hypochondriasis patients. Murad Atmaca et al. reported significantly smaller orbitofrontal cortex (OFC), left thalamus, and pituitary volumes in hypochondriasis patients compared to those in healthy controls (Atmaca et al., 2010, 2016).

Resting-state functional MRI (fMRI) provides distinct information about the functions of brain regions (Zhao et al., 2018b,c). For example, functional connectivity (FC) can reflect the status of integration of local activity between brain regions, which is widely used in psychiatric research (Moreira et al., 2017; Zhao et al., 2018a). However, there is still lack of further investigation of hypochondriasis using resting-state functional connectivity (rs-FC). According to fMRI findings in the literature, altered FC in mental disorders such as schizophrenia, obsessive–compulsive disorder (OCD), and anxiety disorders have been proposed (Liu et al., 2015; Armstrong et al., 2016; Wang et al., 2018; Chen et al., 2019; Cui et al., 2020). The investigation of further FC studies in patients with hypochondriasis is needed.

Support vector machine (SVM) is a classification method successfully applied to diagnostic and prognostic problems (Wenda et al., 2010), which is a supervised learning algorithm popular for its strong theoretical foundation, ability to scale to large datasets, flexibility, and most importantly, accuracy (Chen et al., 2012). Such methods have been used to differentiate patients with psychiatric disorders and healthy controls (Zhu et al., 2018; Li et al., 2019).

This study was conducted to determine potential MRI biomarkers of hypochondriasis and compare patients with hypochondriasis with well-matched healthy controls.

## Materials and Methods

### Participants

In this study, we recruited 21 outpatients with hypochondriasis from the First Affiliated Hospital, College of Medicine, Zhejiang University, Zhejiang Province, China. All patients were diagnosed by at least two professional clinicians using the Structured Interview for DSM-IV Axis I disorders (SCID-I), physical examination and routine laboratory testing. Inclusion criteria for patients to enter this study were as follows: (1) age between 18 and 55 years; (2) a DSM-IV diagnosis of hypochondriasis; (3) being psychiatric drugs naive; (4) negative for HIV antibodies; (5) junior high school education or above; and (6) of Han ethnicity. Patients were excluded if any of the following conditions was met: (1) left-handed; (2) a history or presence of any severe unstable physical disease; (3) substance use disorders or alcohol abuse in the past 6 months; (4) being pregnant, lactating, or planning to become pregnant within the following 6 months; and (5) had metal implants.

Moreover, 22 healthy volunteers without psychiatric disorders matched for hypochondriasis group for age, education, marriage status, and sex were recruited from local communities through advertising.

The Research Ethics Committee of the First Affiliated Hospital, College of Medicine, Zhejiang University, approved the study. All participants and their legal guardians signed the written informed consents.

### Clinical Assessments

The 17-item Hamilton Depression Scale (HAMD-17) was used to assess the severity of depressive symptoms. The Hamilton’ anxiety scale (HAMA) was used to evaluate the severity of anxiety symptoms.

### MRI Data Acquisition

The MRI data were acquired using a Philips Achieva 3.0-T TX MRI system (Philips Healthcare, Netherlands) at the First Affiliated Hospital, College of Medicine, Zhejiang University. The rs-fMRI data were acquired in the axial direction in a sequential mode using a fast field echo-echo-planar imaging (FFE-EPI) sequence, using a head coil with foam padded to strengthen fixation of the head. All participants were instructed to remain still and relaxed, with eyes closed, but awake, clear of any thought.

The rs-fMRI scanning parameters were as follows: 24 slices, repetition time (TR)/echo time (TE) = 2,000/35 ms, flip angle (FA) = 80°, slice thickness/gap = 5.0/1.0 mm, voxel size = 2.4 × 2.4 × 5.0 mm^3^, matrix = 100 × 100, and field of view (FOV) = 240 × 240 mm^2^. The rs-fMRI scan lasted 6 min and 48 s, and we collected a total of 200 image volumes. Individual three-dimensional T1-weighted images were also acquired using the following fast field echo sequence: 150 slices, TR/TE = 7.5/3.7 ms, matrix = 240 × 240, slice thickness = 1 mm, FOV = 240 × 240 mm^2^, voxel size = 1 × 1 × 1 mm^3^, and FA = 8°.

### Imaging Data Preprocessing

The rs-fMRI data were preprocessed using the Advanced DPARSF^1^ and SPM8^2^ toolkits. The first 10 functional volumes were excluded to ensure steady-state longitudinal magnetization. The remaining images were slice-time-corrected based on the middle slice in the time and coregistered to the first image for rigid-body head movement. Images identified with motions greater than 2.0-mm translations or greater than 2.0° rotations at any direction were removed. Consequently, no participants were excluded due to excessive head motion. Next, the linear trend, 24 head-motion covariates, mean white matter, and cerebrospinal fluid (CSF) signals were regressed from each voxel’s time course. Our preprocessing analysis did not perform global signal regression. Subsequently, the images were normalized to the Montreal Neurological Institute space using an EPI template and resampled into 3 × 3 × 3 mm^3^. The data were then smoothed by convolution with an isotropic Gaussian kernel at a full width half maximum (FWHM) of 6 mm and filtered (0.01–0.1 Hz).

### Voxel-Based Morphometry Analysis

This study performed a GMV analysis using a voxel-based morphometry (VBM) method within the SPM8 software package^2^. T1 images were processed using the Montreal Neurological Institute (MNI) template. The whole brain structural data were segmented into white matter, GM, and CSF. Bias correction was applied to remove image intensity non-uniformities. Then, spatial registration was adopted to assess volume changes in segmented GM images. During VBM analysis, the GM volumes were reflected by the modulated images of the GM after bias correction. Finally, GM images were smoothed by applying a Gaussian filter with an 8-mm FWHM kernel.

### Seed-Based FC Analysis

To study the alterations in FC induced by changes in GMV, the brain areas that showed significant differences between the groups in the GMV analysis were selected as the seed regions of interest (ROIs). For each seed ROI, a voxel-wise FC analysis was performed for the preprocessed fMRI data. For each participant and each seed ROI, an FC map of the whole brain was obtained by computing the correlation coefficients between the seeding ROI and the remaining voxels in the entire brain. To improve the normality of the data distribution, the FC maps were converted to z-scores using Fisher’s *r*-to-*z* transformation.

### Statistical Analysis

This study used SPSS (v. 25.0 Chicago, IL, USA) software to perform the statistical analysis for demographic and clinical information. Fisher’s exact test and two-sample *t*-tests were used. For all of the statistical analyses, *p* < 0.05 was considered statistically significant.

To analyze the differences in GMV and FC, we performed voxel-wise two-sample *t*-tests within a GM mask between the hypochondriasis group and healthy control group, with age, sex, head motion, and TIV as covariates (false discovery rate correction, *p* < 0.05).

To examine the correlation between the GMV and FC, a Spearman correlation analysis was performed for each group. The correlation between the GMV or FC results and the clinical variables in hypochondriasis patients were calculated using Spearman’s correlation.

### SVM Classification Analysis

In this study, SVM classifier was tailored to build the predictive model. Both GMV results and FC results were entered into the classification model as feature variables. The *k*-fold cross-validation approach (Abdel-Nasser et al., 2015; Varoquaux et al., 2017), where *k* = 5 was adopted to evaluate the predictive performance of the SVM, was considered as of the small sample size. To investigate the classification accuracy of MR images using GMV and FC as features, a linear kernel SVM was adopted to reduce the risk of overfitting the data, and the weight vector was extracted as an image (i.e., the SVM discrimination map). All the hypochondriasis patients were randomly divided into five subgroups. To fit the parameters of each model, four subgroups were selected, and the left-out subgroup (test set) was used to estimate the hypochondriasis and nonhypochondriasis predictive performance. FeAture Explorer (FAE, v0.2.2^3^) on Python (3.5.4^4^) was used to generate a receiver operating characteristic (ROC) curve, which demonstrated the performance of the SVM model. The accuracy, sensitivity, and specificity were computed for the cutoff value that maximizes the Yorden index. All the above processes were implemented. Both GMV results and FC results were entered into the classification model as feature variables. All the feature variables were normalized and tested for their similarity and removed if pairwise correlations were >0.86; between-group differences.

## Results

### Demographic and Results of the Participants

A total of 21 hypochondriasis outpatients and 22 healthy controls were recruited. Demographic information on age, years of education, and sex, and clinical information regarding HAMD and HAMA cognitive performance scores is presented in Table 1. No significant difference in gender, age, or years of education was noted between the patients and healthy controls. The HAMA score was 18.90 ± 6.12 and HAMD score was 23.29 ± 8.80 in the hypochondriasis group.

**Table 1:** Demographic and clinical information of all participants.

Characteristics	Hypochondriasis (mean ± SD)	HCs (mean ± SD)	*t*/*x*^2^	p
Sample size	21	22
Age (years)	35.43 ± 6.76	31.64 ± 6.06	1.939	0.059
Gender (male/female)	14/7	8/14	0.713	0.095
Years of education (years)	12.86 ± 2.94	13.86 ± 3.41	−1.034	0.307
HAMD	23.29 ± 8.80	0.68 ± 0.65	12.023	0.00
HAMA	18.90 ± 6.12	0.50 ± 0.51	14.056	0.00

*HCs, health controls; HAMD, Hamilton Depression Scale; HAMA, Hamilton Anxiety Scale*.

The hypochondriasis participants all had a history of high-risk sexual behavior and experience of extreme fatigue, low fever, loss of appetite, abdominal distension, diarrhea, coated tongue, and other discomfort after sexual contact. Despite multiple tests showing negative antibodies against HIV and other diseases, they insisted that they were infected a mutated or new, unknown HIV strain. Coincidentally, they were acquainted with each other in a patient QQ group. The exchange of worries and symptoms exacerbated their somatic symptoms and the belief of the same serious illness that they have contracted. Accordingly, they came to our hospital for help together.

### GMV Results

**Figure 1:**
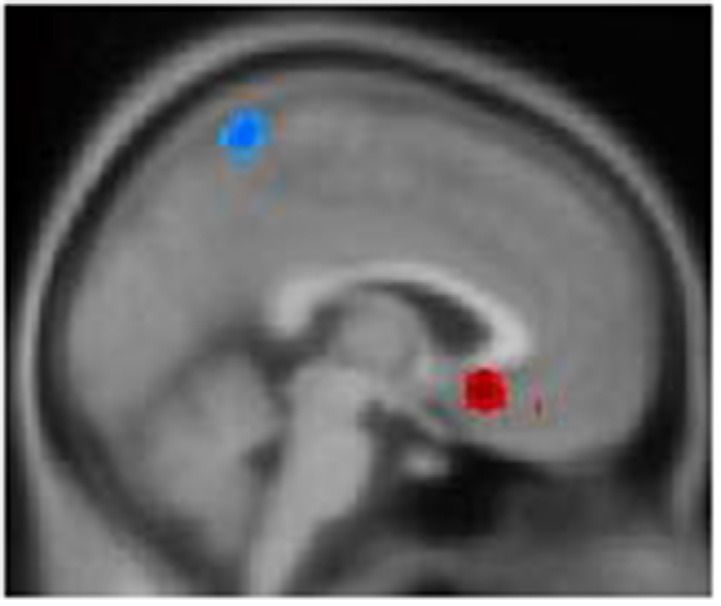
Gray matter volume (GMV) differences among the two groups: the blue color represents decreased volume in hypochondriasis patients, and the red color represents increased volume in hypochondriasis patients compared to controls.

Patients with hypochondriasis showed significantly decreased GMV in left precuneus and increased GMV in the left medial frontal gyrus compared to healthy patients (Table 2, Figure 1).

**Table 2:** Brain regions showing significant GMV and FC difference between hypochondriasis patients and HCs (FDR corrected, *p* < 0.05).

Regions	Hemisphere	Peak MNI			Clusters	*t*-value
		*x*	*y*	*z*		
GMV
Medial frontal gyrus	Left	−4	20	−12	274	5.27
Precuneus	Left	−2	−50	66	130	−4.49
FC
MedFG-cuneus	Left	−9	−87	21	107	−3.96
Precuneus-cuneus	Right	12	−69	3	111	−4.22

*GMV, gray matter volume; FC, functional connectivity*.

### FC Analysis

**Figure 2:**
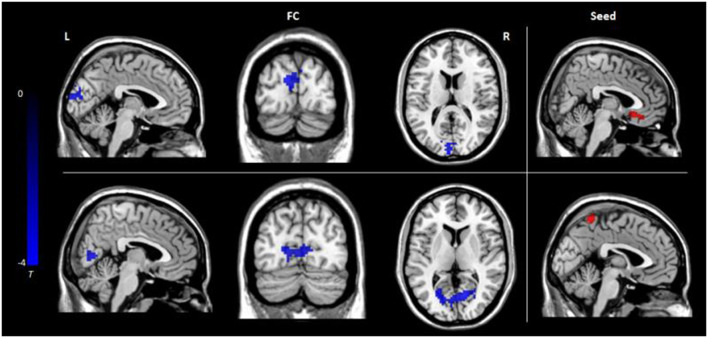
Functional connectivity differences among the three groups: the color bar represents *t* values. L, left; R, right; FC, functional connectivity.

Seed-based FC analyses were performed on ROIs defined in GMV results and revealed decreased FC between the left medial frontal gyrus and cuneus and between the left precuneus and cuneus in the hypochondriasis group relative to that in controls (Table 2, Figure 2).

### Correlation and SVM Analyses

**Figure 3:**
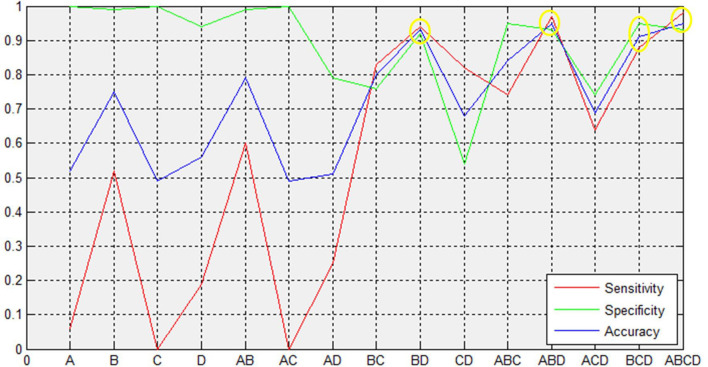
Support vector machine (SVM) results. The combined volume of precuneus and connectivity between precuneus and cuneus could distinguish hypochondriasis from controls accurately. A and B represent the GMV in MedFG and precuneus, respectively. C and D represent the FC between MedFG and cuneus and between precuneus and cuneus, respectively. MFG, medial frontal gyrus; GMV, gray matter volume; FC, functional connectivity; AUC, area under curve; CIs, confidence intervals.

**Figure 4:**
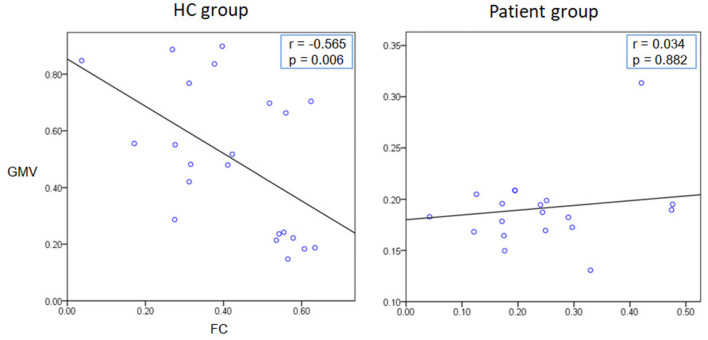
Correlation analysis results. A significant negative correlation was observed between the GMV and FC (*r* = −0.565, *p* = 0.006) among healthy controls.

**Figure 5:**
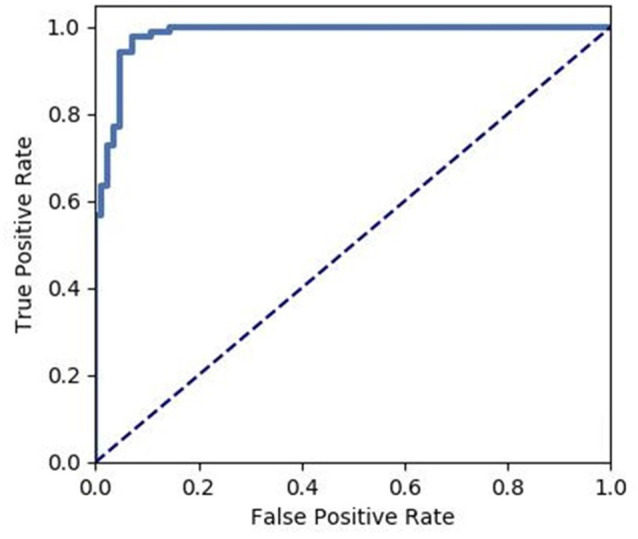
Receiver operating characteristic (ROC) of the discrimination between hypochondriac patients and healthy controls using the GMV and FC values of the significantly different brain regions.

We extracted the average GMV and FC in the brain regions that displayed a significant difference between the two groups. Later, a Spearman correlation analysis was performed between mean GMV and FC across each subgroup. A significant negative correlation was observed between the GMV and FC (*r* = −0.565, *p* = 0.006) among healthy controls (Figure 4). No significant correlation was observed between changes in GMV and HAMD score (MedFG: ρ = −0.177, *p* = 0.442; Precuneus: ρ = −0.09, *p* = 0.70), in FC and HAMD score (Ceunus_MedFG: ρ = 0.254, *p* = 0.266; Ceunus_Precuneus: ρ = 0.310, *p* = 0.172), in GMV and HAMA score (MedFG: ρ = −0.265, *p* = 0.246; Precuneus: ρ = −349, *p* = 0.121), and in FC and HAMA score (Ceunus_MedFG: ρ = 0.014, *p* = 0.953; Ceunus_Precuneus: ρ = 0.420, *p* = 0.058). The final feature variables entered into the classification models were the medial frontal gyrus (A) and precuneus (B) for GMV results, the FC between the medial frontal gyrus and cuneus (C), and between the precuneus and cuneus (D). Table 3 and Figure 3 summarize the area under the curve, sensitivity, specificity, and accuracy for each of these features. However, none of the features discriminated patients with hypochondriasis from healthy controls with satisfactory sensitivity and specificity. We employed SVM analysis to examine whether the combination of the GMV and FC results (features A, B, C, and D) was able to discriminate patients from controls with a sensitivity of 0.98, specificity of 0.93, and accuracy of 0.95 (Figure 5). In addition, the combination of precuneus volume (B) and connectivity between the precuneus and cuneus (D) showed a sensitivity of 0.94, specificity of 0.92, and accuracy of 0.93.

**Table 3:** The results of SVM analysis based on the selected features.

Features	AUC	AUC 95% CIs	Sensitivity	Specificity	Accuracy
A	0.43	0.35–0.52	0.06	1.0	0.52
B	0.79	0.72–0.86	0.52	0.99	0.75
C	0.34	0.26–0.43	0.0	1.0	0.49
D	0.51	0.42–0.60	0.19	0.94	0.56
AB	0.75	0.67–0.83	0.60	0.99	0.79
AC	0.34	0.26–0.42	0.0	1.0	0.49
AD	0.41	0.32–0.49	0.25	0.79	0.51
BC	0.85	0.79–0.90	0.83	0.76	0.80
**BD**	**0.97**	**0.94–0.99**	**0.94**	**0.92**	**0.93**
CD	0.74	0.65–0.81	0.82	0.54	0.68
ABC	0.89	0.84–0.94	0.74	0.95	0.84
**ABD**	**0.98**	**0.96–0.99**	**0.97**	**0.93**	**0.95**
ACD	0.75	0.68–0.82	0.64	0.74	0.69
**BCD**	**0.96**	**0.94–0.99**	**0.88**	**0.95**	**0.91**
**ABCD**	**0.98**	**0.96–0.99**	**0.98**	**0.93**	**0.95**

*A and B represents the GMV in MedFG and Precuneus, respectively. C represent the FC between MedFG and Cuneus, D represent the FC between Precuneus and Cuneus. MedFG, medial frontal gyrus; GMV, gray matter volume; FC, functional connectivity; AUC, area under curve; CIs, confidence intervals; SVM, support vector machine*.

## Discussion

In this study, patients with hypochondriasis all reported a history of high-risk sexual behavior, which triggered concerns of HIV infection. Further, other tests indicated that they were healthy, and they consisted of an unknown virus that infected them. The use of the Internet to search for medical information and communicating in the online community aggravated their feelings of fear and anxiety. A new conception of “cyberchondria” has been invoked, referring to the effects of searching for online medical information (Starcevic, 2017). The Internet cannot always provide accurate and professional information, although patients receive short-term relief from their anxieties after checking, false information enhances their long-term fear and belief of being infected. However, there is still a lack of systematic neuroimaging investigation of those patients.

This study examined whether hypochondriasis patients exhibit different GMV and rs-FC from those in healthy controls. In our study, two brain regions exhibited differences in GMV in hypochondriasis patients, a smaller left precuneus volumes and larger left medial frontal gyrus volumes, from those in healthy controls. Different results were reported in the Atmaca et al. study. They found that gray matter volumes did not differ between patients and healthy controls, and significantly smaller OFC and left thalamus volumes in hypochondriasis, compared to healthy controls (Atmaca et al., 2016). The difference may be due to some reasons: First, different analysis methods, in the Atmaca et al. study, MRI was acquired with a 1.5-Tesla scanner, and the study lacks the segmentation of the gray vs. white matter in the anterior cingulate. Second, demographic variables of age, gender composition, educational level, and duration of illness of patients in the two studies were different, which may lead to different results. Third, the patients in the two studies came from different countries with different cultural backgrounds. Further, we found decreased FC between the left medial frontal gyrus and cuneus and between left precuneus and cuneus, respectively. We discovered that by using SVM analyses, the combination of GMV and FC results can discriminate hypochondriasis from healthy controls. To our knowledge, this is the first study to combine GMV, FC, and SVM to investigate abnormal brain activity in patients with hypochondriasis.

The medial frontal gyrus (MedFG) is part of the medial prefrontal cortex, which is considered to manage the perceptual memory, the extinction of learned fear, and executive function (Kim et al., 2009; Schwiedrzik et al., 2018). Volumetric changes in the MedFG have been reported in adjustment disorder, schizophrenia, and major depression disorder research (Inoue et al., 2012; Frascarelli et al., 2015; Myung et al., 2016; Belleau et al., 2019). The medial frontal cortex is also part of the default mode network (DMN), and mounting evidence suggested that the brain regions in the DMN have an important role in depression and anxiety disorders (Coutinho et al., 2016; Belleau et al., 2019). Although we found no significant correlations between the medial frontal gyrus volume and the severity of depression (HAMD scores) or anxiety (HAMA scores) in patients with hypochondriasis, there is still the possibility that brain connectivity differences are related to some unmeasured factor related to depression or anxiety. On the other hand, we speculate that the increased left medial frontal gyrus volume among patients with hypochondriasis might be associated with executive dysfunction. Many studies have found that deficits in the medial prefrontal cortex are associated with executive dysfunction (Inoue et al., 2012; Cordova et al., 2014). However, the hypochondriasis group showed increased GMV in the left medial frontal gyrus compared to that in healthy controls. Executive function enables the setting of goals and performing goal-directed activities. Increased medial frontal gyrus volume may prompt the enhancement of executive function in patients with hypochondriasis, which could explain their unexplainable beliefs and need for repeated physical examinations, tests, and reassurance from medical professionals. Moreover, the abnormality in this region may explain the overreaction in fear conditioning of hypochondriasis, particularly after high-risk sexual behavior and searching for relevant information online.

The precuneus is involved in self-consciousness (Utevsky et al., 2014), self-related processes (Cavanna and Trimble, 2006), integration of past and present information (Fransson and Marrelec, 2008), and different aspects of memory including episodic memory retrieval (Cavanna and Trimble, 2006) and autobiographical memory (Addis et al., 2004). Considering the specific roles of the precuneus, this finding might reflect a lack of self-related aspects of processing, possibly contributing to hypochondriasis symptoms such as distress from increased negative affect when engaging in excessive Internet searches, overreaction to body signals, and memory distortions.

The results also indicated decreased connectivity of the precuneus and medial frontal gyrus with the cuneus in patients with hypochondriasis. The cuneus is functionally connected to a visual network and is well known in basic visual processing (Vanni et al., 2001). The precuneus and medial frontal gyrus are both major nodes of the main functional and structural networks of the human brain, with a relevant role within the default mode network (DMN; Utevsky et al., 2014). The DMN has been found to be involved in inwardly focused mental processes such as self-referential processing, personal introspection, autobiographical memory, and future thinking, all of which contribute to a coherent sense of self (Bluhm et al., 2009; Liemburg et al., 2012; Sripada et al., 2012; Lanius et al., 2015; Pankow et al., 2015). However, there are no reported DMN abnormalities in hypochondriasis. In addition, an fMRI study reported a decreased recruitment of the precuneus, caudate nucleus, globus pallidus, and thalamus in patients with hypochondriasis, compared with that in healthy controls, which suggests the dysfunction of the precuneus and thalamus in hypochondriasis (van den Heuvel et al., 2011). Hypochondriasis was classified in the Obsessive–Compulsive and Related Disorders in ICD-11 (van den Heuvel et al., 2014; Stein et al., 2016). Meanwhile, similar brain abnormalities have been found in patients with obsessive–compulsive disorder (OCD). It has been suggested that hypochondriac patients behave like OCD patients with respect to brain abnormalities and pathophysiology (Atmaca et al., 2016). Several studies have reported alterations in brain connectivity within the DMN in OCD (Goncalves et al., 2017). In the present study, the hypochondriasis group showed decreased GMV in the left precuneus and increased GMV in the left medial frontal gyrus compared with those in the healthy control group, and decreased FC between the left medial frontal gyrus and cuneus, and between the left precuneus and cuneus, which emphasize dysfunction in the DMN of hypochondriasis. As a consequence, hypochondriasis may be attributable to the abnormalities of DMN, and it may be one of the primary reasons for patients to pay too much attention to their body signals and insist they are ill. Further studies are needed to investigate other brain regions involved in the DMN in hypochondriasis.

The SVM analysis suggested that a combination of both GMV and FC in the left precuneus, medial frontal gyrus, and cuneus was able to discriminate the hypochondriasis patients from healthy controls with a sensitivity of 0.98, specificity of 0.93, and accuracy of 0.95. Interestingly, an optimum combination can distinguish hypochondriasis from heathy controls. Serving as the most remarkable findings of this study, the combination of precuneus volumes and FC in the left precuneus and cuneus for correctly classifying patients had a specificity of 0.94, sensitivity of 0.92, and accuracy of 0.93, which suggests that the left precuneus and cuneus may be potential imaging biomarker in hypochondriasis.

Overall, our study has some limitations. First, the number of subjects was relatively small, which may have caused default negative results. Although the performance of the predictive model was tested using a k-fold cross validation method, independent samples are needed to train and test the model. A larger sample size might be a way to increase the reliability in the future. Second, it is unfortunate that the rating scale for hypochondriac symptoms was not evaluated. In future studies, hypochondriasis symptoms should be assessed. Third, the correlations between the brain regions that exhibited differences and mood symptoms were not distinguished. Therefore, which changes contribute to depressive or anxious mood remain speculative. Future studies based on a prospective design and larger sample size can contribute to a better understanding of the mechanism of hypochondriasis.

To conclude, the results from the current investigation suggest that hypochondriasis patients had a smaller left precuneus volumes and decreased FC between left precuneus and cuneus compared with those in healthy controls. This dysfunction seems to play an important role in the pathophysiology of hypochondriasis.

## Data Availability Statement

The raw data supporting the conclusions of this article will be made available by the authors, without undue reservation.

## Ethics Statement

The studies involving human participants were reviewed and approved by Research Ethics Committee of the First Affiliated Hospital, College of Medicine, Zhejiang University. The patients/participants provided their written informed consent to participate in this study.

## Author Contributions

ZS, LY, MH and ZZ participated in the design of this study, and they all performed the statistical analysis. ZS and LY carried out the study and collected important background information. ZS drafted the manuscript. KJ, FP, SH and SL provided assistance for literature search, data acquisition and data analysis. DX, YX and MH performed manuscript review. All authors read the final manuscript. All authors contributed to the article and approved the submitted version.

## Conflict of Interest

The authors declare that the research was conducted in the absence of any commercial or financial relationships that could be construed as a potential conflict of interest.

## Footnotes

^1^http://resting-fmri.sourceforge.net

^2^http://www.fil.ion.ucl.ac.uk/spm

^3^https://github.com/salan668/FAE

^4^https://www.python.org
